# First evidence of pyrethroid resistance in Italian populations of West Nile virus vector *Culex pipiens*

**DOI:** 10.1111/mve.12573

**Published:** 2022-04-09

**Authors:** Verena Pichler, Carola Giammarioli, Romeo Bellini, Rodolfo Veronesi, Daniele Arnoldi, Annapaola Rizzoli, Riccardo Paolo Lia, Domenico Otranto, Marco Ballardini, Pietro Cobre, Paola Serini, Alessandra della Torre, Beniamino Caputo

**Affiliations:** ^1^ Dipartimento di Sanità Pubblica e Malattie Infettive Università Sapienza Rome Italy; ^2^ Centro Agricoltura Ambiente “G. Nicoli”, Department of Medical and Veterinary Entomology Crevalcore Italy; ^3^ Ecohealth Unit, Research and Innovation Centre Fondazione Edmund Mach, San Michele all'Adige Trento Italy; ^4^ Dipartimento di Medicina Veterinaria Università di Bari Valenzano Italy; ^5^ Istituto Zooprofilattico Sperimentale del Piemonte Liguria e Valle d'Aosta Torino Italy

**Keywords:** *Culex pipiens*, Europe, insecticide resistance, pest management, vector control

## Abstract

*Culex pipiens* (Linnaeus), one of the most abundant mosquito species in Europe, plays a crucial role in the endemic transmission of West Nile virus and caused the large outbreak with >1600 human cases in 2018. Although evidence of resistance to pyrethroids has been reported for *Cx. pipiens* populations from Spain and Greece, resistance monitoring has been largely neglected in Italy. Herein, we investigate susceptibility of Italian *Cx. pipiens* populations to the pyrethroids permethrin and deltamethrin. Results from WHO‐tube‐bioassays revealed mortalities ranging from 14–54%, indicating high levels of resistance, in four out of 10 populations exposed to permethrin (0.75%) and of 63% in one of three populations exposed to deltamethrin (0.05%). Reduced susceptibility (mortality<98%) was detected in almost all other populations. A clear association is shown between the resistant phenotype and the presence of *kdr*‐alleles in position 1014 of the VSSC, strongly suggesting its role in reducing susceptibility. The study provides the first evidence of pyrethroid‐resistance in Italian *Cx. pipiens* populations and reports levels of resistance paralleled in the European region only in Turkey. This highlights the urgent need to implement insecticide‐resistance management plans to restore the efficacy of the nowadays only chemical weapon available to control arbovirus transmission in Europe.

## Graphical abstract

We report the first evidence of Pyrethroid resistance in Italian *Culex pipiens* populations.

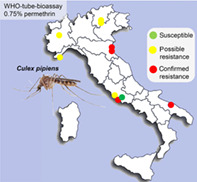

The resistance phenotype appears to be substantial and widespread possibly affecting the effectiveness of pyrethroid treatments in case of arbovirus outbreaks.

The *kdr*‐mutation L1014F within the VSSC appears to be one of the mechanisms underlying resistance.

## INTRODUCTION

*Culex pipiens* (Linnaeus) is one of the most abundant mosquito species in Europe and Italy (Mancini et al., 2017; Severini et al., 2009) and plays a crucial role in the transmission of three arboviruses currently circulating in Europe: West Nile virus (WNV), Usutu virus (USUV) and Sindbis virus (SINV), with WNV having the highest epidemiological impact (Brugman et al., 2018). WNV is usually transmitted in an enzootic cycle involving birds and mosquitoes. Humans and horses can occasionally get infected (dead‐end hosts) and in some cases develop a neuro‐invasive form with a 10–20% fatality rate (Brugman et al., 2018). While the disease was considered mainly a tropical arbovirosis until the 1990s, in the last decades, an increasing number of human cases was reported also in temperate regions with the largest outbreak in Europe occurring in 2018, resulting in more than 1600 confirmed human cases, most of which in Italy, Greece, Romania and Hungary (Brugman et al., 2018, https://atlas.ecdc.europa.eu/public/index.aspx).

In addition to the public health burden, WNV transmission has also a significant economic impact, due to direct costs linked to the human illness in the short term (estimated in Italy as >15,000€ for hospital care for each case of neuro invasive disease [Paternoster et al., 2017]), as well as to indirect costs. These include primarily the screening of blood donations for WNV presence in order to prevent transmission by blood transfusion. For the sole Emilia Romagna (north‐east Italy), a region particularly affected by WNV circulation, such indirect costs are estimated to exceed 400,000€ per year (Paternoster et al., 2017).

At present no specific treatment nor vaccine against WN disease in humans is available: therefore, preventive measures to reduce human‐vector contact (e.g., appropriate clothing, use of repellents, window screens) and control interventions targeting *Cx. pipiens* are the only tools available to reduce disease transmission (ECDC, 2020). In agreement with the European Centre for Disease Control guidelines, the Italian Ministry of Health recommends to prevent high mosquito densities and nuisance by targeting the larval developmental stage (Ministero della Salute, 2019). Interventions targeting adult mosquitoes by ground‐spraying of pyrethroids – the only insecticidal class authorized for the control of adult mosquitoes in EU (EU Directive 528/2012, n.d.) – are only recommended in case of ongoing disease transmission, when a fast abatement of adult mosquitoes – able to transmit WNV – is necessary. However, the often uncontrolled and incorrect usage of pyrethroids to reduce mosquito nuisance and to control agricultural pests in Europe creates the condition for the development and spread of pyrethroid resistance (PR) in *Cx. pipiens*, as already largely observed for other mosquito vectors worldwide (Rinkevich et al., 2013).

Differently from main vector species – such as anopheline mosquitoes or *Aedes aegypti* – knowledge on PR in *Cx. pipiens* is limited, and no official WHO protocols for the evaluation of phenotypic resistance in this species exist, making it thus difficult to compare data between studies. In the European Union, phenotypic resistance to two of the most commonly used pyrethroids, permethrin and deltamethrin, has been so far investigated and recorded only in *Cx. pipiens* populations from Spain (Paaijmans et al., 2019) and Greece (Fotakis et al., 2017; Kioulos et al., 2013).

Target‐site mutations are among the main mechanisms underlying PR in mosquitoes. Pyrethroids target the voltage‐sensitive sodium channel (VSSC) involved in correct nervous signal transmission, interfere with the channel kinetics and lead to a fast knockdown and eventually death of the mosquito (Hemingway & Ranson, 2000). The presence of non‐synonymous mutations in the VSSC (known as knock‐down resistance‐ or *kdr*‐mutations) can hinder the binding of pyrethroids, lowering thus the susceptibility of mosquitoes to these compounds (Hemingway & Ranson, 2000; Rinkevich et al., 2013). In Europe, two mutations in position 1014 of the VSSC – L1014F and L1014C – have been detected so far in *Cx. pipiens* populations from Greece (Fotakis et al., 2017; Kioulos et al., 2013).

Although Italy is one of the European countries most heavily impacted by WN disease, no information on either the phenotypic resistance status of Italian *Cx. pipiens* nor the circulation of *kdr* alleles in this species is available. This is even more concerning when considering that pyrethroid‐resistant *Aedes albopictus* (Skuse) populations sympatric to *Cx. pipiens* have been detected (Pichler et al., 2018, 2019). The present study aims at tackling this knowledge gap by investigating susceptibility of Italian *Cx. pipiens* populations to two of the most used pyrethroids in the country (i.e., permethrin and deltamethrin) from both, a phenotypic and genotypic perspective.

## MATERIALS AND METHODS

### Mosquito collections and rearing

Collections of *Cx. pipiens* egg rafts or larvae were carried out in 2016 and 2017 from June to November in 10 sites from 7 Italian provinces; in six of these sampling sites, pyrethroid treatments were carried out regularly during the sampling season (Table S1). Eggs or larvae were picked up directly from larval breeding sites such as road ditches and sewers by local entomology teams and at least 3–5 different breeding sites at 100 m distance were sampled in each site to avoid oversampling of siblings. Sampled eggs were stored on wet filter paper, and larvae were maintained in plastic containers filled with water and then sent by express courier to the Department of Public Health and Infectious Diseases (DPHID) at Sapienza University of Rome.

Larvae were reared at larval density of 100 larvae/l in the insectary of DPHID at T = 26 ± 1°C, RH = 60 ± 5%, 14:10 h light: dark photoperiod and fed with artificial dry cat‐food. Pupae were collected daily and transferred into 40 cm^3^ cages. Emerged adults were identified as *Cx. pipiens* using morphological keys (Severini et al., 2009) and kept at the same temperature and humidity as larvae and fed with 5% sugar solution at libitum.

### Insecticide susceptibility bioassays

Bioassays were performed according to WHO protocols (World Health Organization, 2016) in WHO test tubes lined with filter papers impregnated with permethrin (0.75%) or deltamethrin (0.05%) (Vector Control Research Unit, School of Biological Sciences, 11800 Minden, Penang, Malaysia). Since no official WHO guidelines for bioassays on *Cx. pipiens* exist, insecticide concentrations were selected based on dosages for which a 100% mortality was reported for different susceptible laboratory strains and which were most frequently used in published articles on *Cx. pipiens* to allow a comparison with results from previous studies (Table S2).

Insecticide impregnated (and control) papers were discarded after being used in 6 bioassays as recommended by WHO. Quality of test papers was confirmed by performing in parallel bioassays on a susceptible *Ae. albopictus* population (more than >8o generations in lab), which showed 100% mortality to both tested insecticides.

Bioassays were performed in the insectary at the same conditions of mosquito rearing on 3‐ to 5‐day old unfed *Cx. pipiens* females emerged from field collected eggs/larvae (F0). Each replicate was performed on 20–25 specimens/tube, and a total of 3–4 replicates/site/pyrethroid were performed for most populations, as recommended by WHO (World Health Organization, 2016). The number of knocked down mosquitoes (i.e., mosquitoes unable to stand or fly in a coordinated way) was recorded every 10 min during exposure time. After 1 h of exposure, mosquitoes were transferred into tubes with untreated papers and allowed a 24‐h recovery, after which they were classified as either dead or survived. Control tubes (i.e., tubes lined with filter papers impregnated only with the insecticide excipient, but without the active ingredient) were set up and handled as the test tubes.

Mean values of mortality and 95% confidence intervals were computed for each population. When mortality in control cages was between 5% and 20% Abbott's correction for natural mortality was applied. Populations were considered ‘susceptible’ if mortality at 24 h after exposure was ≥98%, ‘possibly resistant’ if mortality ranged between 80% and 98% and ‘resistant’ if mortality was <80%. We decided to maintain the 80% threshold (as recommended by WHO, 1998) to define populations as resistant for a more conservative analysis of the results, even though other protocols recommend a threshold of 90%. For knock‐down assessment, a log time‐probit statistical model was applied to compute KD curves for each population and to calculate 50% (KDT50) and 95% (KDT95) knockdown times. Pearson's correlation coefficient was computed to evaluate correlation between KDT values and percentage mortality. All analysis were carried out using R software version 3.3.3; the R‐script used for computation can be provided by the authors upon request.

### VSSC resistance mutation genotyping

Genomic DNA was extracted from a subset of mosquitoes previously exposed to permethrin (selected from the 10 sampled populations), using the DNAzol (MRC. Inc., Cincinnati, Ohio) reagent following manufacturer's instructions. Genotyping of mutations in position 1014 of the VSSC protein was carried out by the allele‐specific PCR (AS‐PCR) described by Martinez‐Torres et al. (1999). While this AS‐PCR approach was designed to detect the 1014F allele, it was shown to produce the same banding pattern also in presence of allele 1014C (Fotakis et al., 2017). Thus, since this AS‐PCR does not allow to discriminate between the two mutations, in the results, we will refer to resistant (i.e., 1014F or 1014C) and susceptible (i.e., 1014L) alleles, only.

PCR products were visualized by electrophoresis on a 2% agarose gel stained with Midori Green Advance (Nippon Genetics, Tokyo, Japan) and visualized under UV light. Association between the detected genotypes and permethrin susceptibility was evaluated using a chi‐square test.

## RESULTS

### Insecticide susceptibility bioassays

**FIGURE 1:**
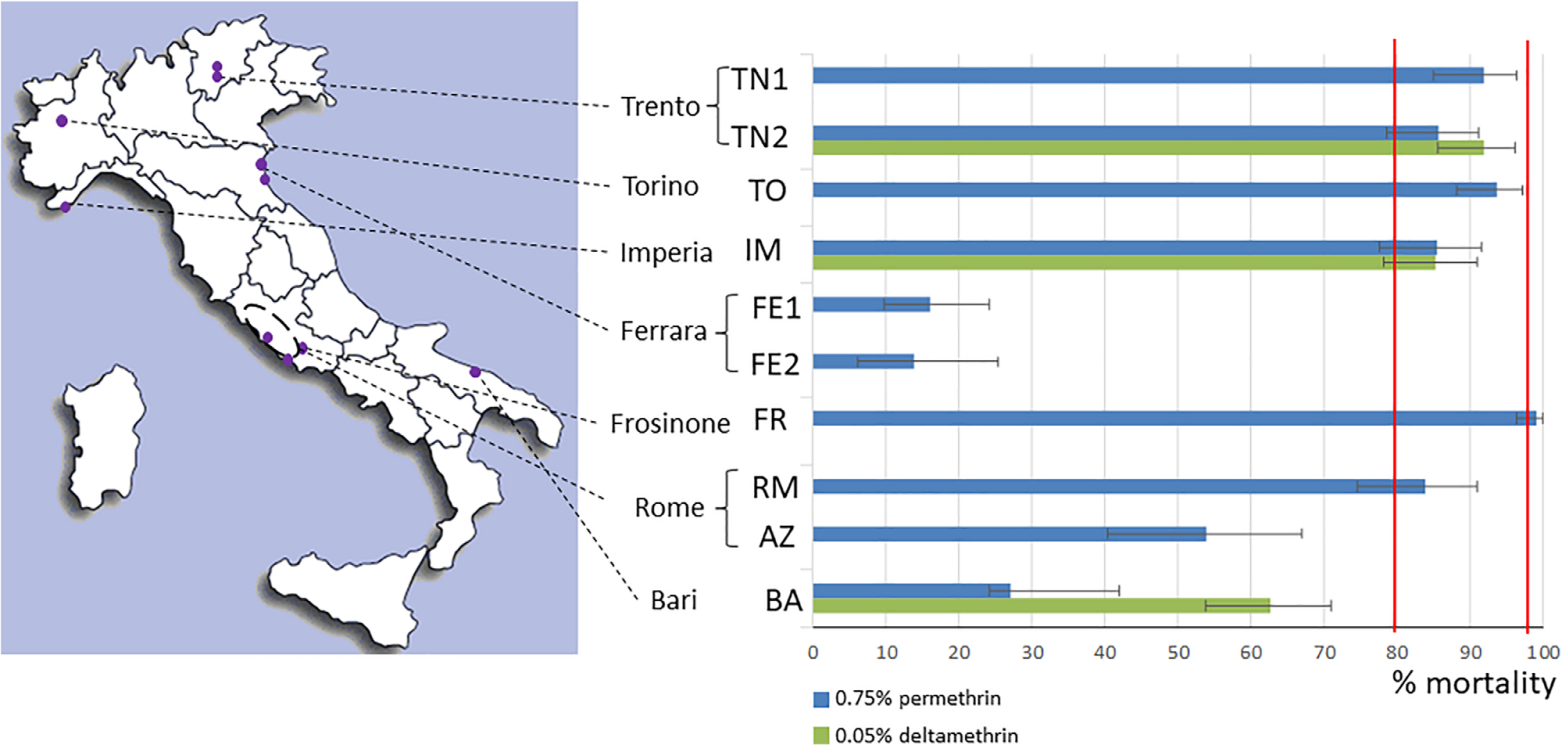
Distribution of tested *Culex pipiens* adult populations in Italy and mortality (%) at 24 h after 1 h exposure to either 0.75% permethrin (blue) or 0.05% deltamethrin (green) in WHO test tubes. Vertical lines indicate 80% and 98% mortality thresholds. Horizontal whiskers show ±95% confidence intervals for mortality

Susceptibility and KDTs to permethrin (0.75%) and deltamethrin (0.05%) were assessed in 10 and 3 *Cx. pipiens* populations, respectively (Figure 1, Table S1).

#### Permethrin

Bioassays in WHO test tubes were performed for 10 populations from 7 Italian provinces (*N* = 912 specimens) (Figure 1). Mortality ranged from 14% (Ferrara: FE2) to 99.2% (Frosinone: FR), with only the FR population from Frosinone province showing full susceptibility. Mortality ranging from 84–93.7%, suggesting possible resistance, was observed for five populations from Trento, Torino, Imperia and Rome provinces (TN1, TN2, TO2, IM and RM). Mortality clearly below 80% indicating high levels of resistance was detected in Ferrara (FE1: 16.2% and FE2: 14%), Rome (AZ: 53.8%) and Bari provinces (BA: 27.1%). Consistent with mortality results, KDT values (Figure S1) were highest for populations from Ferrara, Bari and Rome provinces and lowest for the FR population, and correlation between KDT50, KDT95 and mortality was significant (r_KDT50/mortality_ = −0.92, d.f. = 8, *p*‐value < 0.01; r_KDT95/mortality_ = −0.71, d.f. = 8, *p*‐value < 0.02).

#### Deltamethrin

Bioassays in WHO test tubes were performed for 3 populations from Trento (TN2), Imperia (IM) and Bari (BA) provinces, respectively (*N* = 334 specimens) (Figure 1, Table S1). None of these displayed full susceptibility to deltamethrin. Mortality observed for populations from Trento and Imperia provinces indicated possible resistance (mortality: TN2 = 92%: IM = 85.3%), while mortality for the population from Bari suggested resistance to this compound (BA = 62.71%). KDT values (Figure S1) are consistent with these results and range from 14.3 (TN2) to 36.8 (BA) minutes for KDT50 and from 60.5 (TN2) to 140.8 (BA) for KDT95.

### VSSC genotyping

PCR genotyping of *kdr*‐mutations in position 1014 of the VSSC was successful for 121 out of 136 mosquitoes. Both, dead (*N* = 47) and 0.75% permethrin bioassay survivors (*N* = 74) were included in the analyses (Tables 1 and S3). Resistance alleles in position 1014 were detected in all the 10 populations with highest frequencies in sites from Ferrara province (Table S3). A significant association between the presence of *kdr‐*alleles and permethrin susceptibility was observed (*χ* ^2^ = 32.77; *p*‐value < 0.0001; d.f. = 1).

**TABLE 1:** Genotype and allele frequency for resistant (R) and susceptible (S) alleles in position 1014 of the VSSC and total number of *Culex pipiens* specimens genotyped, grouped per permethrin (0.75%) WHO tube bioassays outcome (dead or survivor)

Bioassay outcome	Total (N) genotyped	1014F genotype			Freq (R)
		RR	RS	SS	
Dead	47	0.19	0.43	0.38	0.40
Survivor	74	0.68	0.20	0.12	0.78
TOTAL	121	0.49	0.29	0.22	0.63

## DISCUSSION

Data herein presented highlight the existence of Italian *Cx. pipiens* populations highly resistant to permethrin (0.75%) and deltamethrin (0.05%), as well as a widespread reduced sensitivity to both compounds in all populations under investigation, except one. This almost complete lack of effectiveness of pyrethroids is of concern considering the importance of this mosquito species as vector of WNV.

The lack of a reference *Cx. pipiens* susceptible colony may represent a limitation inherent to the study design, since it could have further confirmed the effectiveness of insecticide‐impregnated filter papers. Anyway, the effectiveness of the used WHO filter paper was supported by the presence in our experiments of a population showing >99% mortality, as well as the 100% mortality observed on a susceptible *Ae. albopictus* population tested in parallel bioassays. In addition, in order to avoid loss of selective pressure and inbreeding under laboratory conditions, we chose to perform bioassays on F0 females, which irremediably implied that in few cases we could not test a minimum of 100 females per insecticide, as recommended by WHO to confirm resistance (World Health Organization, 2016).

Analysis of a susceptible laboratory colony could have greatly facilitated the interpretation of our results since WHO does not provide specific diagnostic dosages for *Cx. pipiens*. Anyway, we used the 0.75% permethrin and 0.05 deltamethrin concentrations in the bioassays as different studies (Table S2) reported a 100% mortality for susceptible laboratory strains, confirming the suitability of these concentrations. Moreover, being these the most frequently used dosages for WHO tube assays, they allow a comparison of results herein obtained with previous studies. Among studies applying the same approach, levels of resistance as high as in the present study – and thus suggesting almost complete lack of effectiveness of the tested pyrethroid – have been so far detected only by WHO bioassays on Turkish populations, where Guntay et al. (2018) reported mortalities <10% for all populations exposed to permethrin.

A precise assessment of pyrethroid usage and thus the relative role of mosquito control as selective pressure for the insurgence of insecticide resistance in sampling sites is difficult. Indeed, pyrethroid usage is highly heterogeneous due to a variety of factors in applications (e.g., differences in dosages, spraying methods, protocols and time schedules) as well as the occurrence of concomitant treatments at the public and private level not only for mosquito control but also for other insect pests. Despite this, an interesting PR distribution pattern was recorded: *Cx. pipiens* populations from sites where pyrethroid treatments against mosquitoes are implemented only rarely (≤ once/year) showed mortality >85% in Trento and Imperia provinces (TN1, TN2, IM) and > 99% in FR site (Frosinone province). On the other hand, lowest mortality is observed in populations from coastal sites in AZ (Rome province), BA (Bari province), FE1 and FE2 (Ferrara province). Notably, also *Ae. albopictus* populations in these same sites showed clear signs of resistance to permethrin (Pichler et al., 2018, 2019), corroborating the hypothesis that heavy pyrethroid spraying – largely exploited during the last few decades to reduce mosquito nuisance in these touristic sites – is responsible for the observed reduction in susceptibility. Indeed, the two localities from Ferrara province are highly touristic sites along the Adriatic coast in Comacchio area, where insecticide spraying has been widely used since 1991 to reduce nuisance during summer seasons (Bellini & Veronesi, 1994). Interestingly, these treatments usually did not target primarily *Cx. pipiens*, but other mosquito species particularly annoying to humans due to their aggressive and daytime biting behaviour, such as *Ae. caspius* (Pallas) or *Ae. albopictus*. The reduction in susceptibility observed herein for *Cx. pipiens* appears to be higher compared to *Ae. albopictus* (Pichler et al., 2018, 2019), whereas no data on Italian *Ae. caspius* populations exist. While we cannot exclude that at least part of this difference of the resistance phenotype is due to a different baseline susceptibility to permethrin of the two species, another possible explanation could be the way pyrethroid treatments are implemented. In fact, they are often performed at night to reduce nuisance to human populations (RB, personal communication), causing a higher level of exposure in *Cx. pipiens*, which is active night‐times. Moreover, since *Cx. pipiens* is endemic in Italy – as opposed to invasive *Ae. albopictus* – it is possible that it has been exposed to selective pressure by pyrethroid treatments for more years, thus reaching a higher level of PR. It is also relevant to note that other studies reported also reduced susceptibility to the larvicide diflubenzuron (DFB), as well as the circulation at high frequency (>60%) of several mutations conferring resistance to DFB, in *Cx. pipiens* populations from Ferrara and other neighbouring provinces (Grigoraki et al., 2017; Porretta et al., 2019).

The clear association we detected between the presence of *kdr‐*alleles in position 1014 and the probability to survive the bioassays suggests a role of these mutations in conferring resistance, though other mutations or metabolic resistance factors cannot be ruled out. In fact, 15% of individuals homozygous for the resistance alleles – and thus supposed to express the strongest resistance phenotype given the probable recessive mode of the *kdr* mutation inheritance (Hemingway & Ranson, 2000) – died during the bioassay, while 33% of homozygotes for the susceptible allele 1014 L survived. In Europe the circulation of mutations in position 1014 at frequencies >60% was already detected in Greek populations (Fotakis et al., 2017) and both alleles, 1014F and 1014C, were identified as factors responsible for PR. Due to the low number of specimens analysed per site, observed differences in frequency of *kdr* mutations between populations need to be taken with caution and to be confirmed in future studies. Further assessments will be necessary also to identify which resistance alleles are circulating in Italy, as well as to understand their emergence/spread and how much different alleles contribute to the PR phenotype. In addition, concurrence of metabolic resistance mechanisms in conferring resistance has already been reported in Greece (Fotakis et al., 2017) and Turkey (Guntay et al., 2018). Further investigations are needed to clarify whether these metabolic mechanisms contribute to resistance also in Italian populations.

Overall, the picture emerging from this and few other studies (Grigoraki et al., 2017; Porretta et al., 2019) on insecticide resistance in Italian *Cx. pipiens* populations ‐ with evidence of widespread reduced susceptibility to pyrethroids and highest levels of both PR‐ and DFB‐resistance in coastal touristic sites ‐ highlights the urgent need to reduce pyrethroid usage for nuisance reduction in order to restore/maintain the efficacy of the only chemical tool available to stop/reduce arbovirus transmission. Effective insecticide resistance management plans, including evaluation of changes in PR levels by monitoring periodically phenotypic and genotypic resistance in vector species, need to be implemented in order to oppose the possible insurgence and spread of multi‐resistant mosquitoes.

## CONFLICT OF INTEREST

The authors declare no conflict of interest.

## AUTHOR CONTRIBUTIONS

Verena Pichler, Beniamino Caputo, Alessandra Della Torre conceived the study. Verena Pichler, Carola Giammarioli, Beniamino Caputo, Romeo Bellini, Rodolfo Veronesi, Daniele Arnoldi, Annapaola Rizzoli, Riccardo Paolo Lia, Domenico Otranto, Marco Ballardini, Pietro Cobre, Paola Serini performed sample collection and experiments. Verena Pichler performed data analysis and wrote the first draft of the manuscript. All authors reviewed and approved the final manuscript.

## Supporting information

Supplementary Material 1 :
**Table S1.** Results of WHO tube bioassays performed on *Cx. pipiens* adult populations from 10 sites in 7 Italian provinces exposed to permethrin (0.75%) and deltamethrin (0.05%). Number of tested females are reported, as well as mortality (%, ±95% confidence intervals CI) at 24 h after a 1‐h exposure and times to knock‐down in minutes (KDT) of 50% and 95% of the population (with 95% confidence intervals [CIs]).
**Table S3.** Genotype and allele frequency for resistant (R) and susceptible (S) alleles in position 1014 of the VSSC and total number of *Cx. pipiens* specimens genotyped, grouped per sampling site.
**Figure S1.** Knock‐down times (and 95% confidence intervals) of 50% (KDT50; blue) and 95% (KDT95; yellow) of Italian *Cx. pipiens* populations exposed to permethrin (0.75%) and deltamethrin (0.05%).

Supplementary Material 2:
**Table S2.** Details on articles reporting WHO bioassay data for *Culex pipiens* mosquitoes exposed to deltamethrin and/or permethrin.

## Data Availability

All data produced within the present study are available within the article and its supplementary material.
