# Supplementary material for: First evidence of pyrethroid resistance in Italian populations of West Nile virus vector Culex pipiens
Source: Med Vet Entomol. 2022 Apr 9;36(3):390–5. doi: 10.1111/mve.12573 (PMC9540436; doi:10.1111/mve.12573)
Supplement: Supplementary file 1 — Supplementary Material 1 : Table S1. Results of WHO tube bioassays performed on Cx. pipiens adult populations from 10 sites in 7 Italian provinces exposed to permethrin (0.75%) and deltamethrin (0.05%). Number of tested females are reported, as well as mortality (%, ±95% confidence intervals CI) at 24 h after a 1‐h exposure and times to knock‐down in minutes (KDT) of 50% and 95% of the population (with 95% confidence intervals [CIs]). Table S3. Genotype and allele frequency for resistant (R) and susceptible (S) alleles in position 1014 of the VSSC and total number of Cx. pipiens specimens genotyped, grouped per sampling site. Figure S1. Knock‐down times (and 95% confidence intervals) of 50% (KDT50; blue) and 95% (KDT95; yellow) of Italian Cx. pipiens populations exposed to permethrin (0.75%) and deltamethrin (0.05%). [file MVE-36-390-s002.docx]

**First evidence of pyrethroid resistance in Italian populations of West Nile virus vector *Culex pipiens***

Verena Pichler^1^, Carola Giammarioli^1^, Romeo Bellini^2^, Rodolfo Veronesi^2^, Daniele Arnoldi^3^, Annapaola Rizzoli^3^, Riccardo Paolo Lia^4^, Domenico Otranto^4^, Marco Ballardini^5^, Pietro Cobre^1^, Paola Serini^1^, Alessandra della Torre^1^, and Beniamino Caputo^1^

1.Dipartimento di Sanità Pubblica e Malattie Infettive, Università Sapienza, Rome, Italy;

2.Centro Agricoltura Ambiente “G. Nicoli”, Crevalcore, Italy;

3.Ecohealth Unit, Research and Innovation Centre, Fondazione Edmund Mach, San Michele all’Adige, Trento, Italy;

4.Dipartimento di Medicina Veterinaria, Università di Bari, Valenzano, Italia;

5.Istituto Zooprofilattico Sperimentale del Piemonte, Liguria e Valle d’Aosta, Torino, Italy

## Supplementary Material

**Table S1. Results of WHO tube bioassays performed on Cx. pipiens adult populations from 10 sites** in 7 Italian provinces exposed to permethrin (0.75%) and deltamethrin (0.05%). Number of tested females are reported, as well as mortality (%, ± 95% confidence intervals CI) at 24 h after a 1-h exposure and times to knock-down in minutes (KDT) of 50% and 95% of the population (with 95% confidence intervals CIs).

a= Abbott corrected values

†= pyrethroid treatments performed during sampling season

**Table S3. Genotype and allele frequency for resistant (R) and susceptible (S) alleles** in position 1014 of the VSSC and total number of *Cx. pipiens* specimens genotyped, grouped per sampling site.

|  |  |  | **Total (N) genotyped** | **1014F genotype** | | | **Freq (R)** |
| --- | --- | --- | --- | --- | --- | --- | --- |
| **province** | **site-code** | **site** |  | **RR** | **RS** | **SS** |  |
| **Trento** | TN1 | San Michele | 7 | 0.43 | 0.00 | 0.57 | 0.43 |
|  | TN2 | Zambana | 24 | 0.17 | 0.58 | 0.25 | 0.46 |
| **Torino** | TO2 | Torino | 12 | 0.08 | 0.75 | 0.17 | 0.46 |
| **Imperia** | IM | Imperia | 11 | 0.82 | 0.18 | 0.00 | 0.91 |
| **Ferrara** | FE1 | Lido di Volano | 18 | 0.89 | 0.06 | 0.06 | 0.92 |
|  | FE2 | Lido di Spina | 9 | 1.00 | 0.00 | 0.00 | 1.00 |
| **Frosinone** | FR | Strangolagalli | 1 | 0.00 | 1.00 | 0.00 | 0.50 |
| **Rome** | RM | Roma | 11 | 0.00 | 0.09 | 0.91 | 0.05 |
|  | AZ | Anzio | 14 | 0.43 | 0.29 | 0.29 | 0.57 |
| **Bari** | BA | Bari | 14 | 0.79 | 0.21 | 0.00 | 0.89 |

**Figure S1. Knock-down times (and 95% confidence intervals) of 50% (KDT50; blue) and 95% (KDT95; yellow) of Italian Cx. pipiens** populations exposed to permethrin (0.75%) and deltamethrin (0.05%).
